# Development of cassava core collections based on morphological and agronomic traits and SNPS markers

**DOI:** 10.3389/fpls.2023.1250205

**Published:** 2023-09-06

**Authors:** Caroline Cardoso dos Santos, Luciano Rogerio Braatz de Andrade, Cátia Dias do Carmo, Eder Jorge de Oliveira

**Affiliations:** ^1^ Centro de Ciências Agrárias, Ambientais e Biológicas, Universidade Federal do Recôncavo da Bahia, Cruz das Almas, Bahia, Brazil; ^2^ Embrapa Mandioca e Fruticultura, Nugene, Cruz das Almas, Bahia, Brazil

**Keywords:** *Manihot esculenta* Crantz, germplasm, breeding, genetic resources, SNP

## Abstract

Cassava (*Manihot esculenta* Crantz) holds significant importance as one of the world’s key starchy crop species. This study aimed to develop core collections by utilizing both phenotypic data (15 quantitative and 33 qualitative descriptors) and genotypic data (20,023 single-nucleotide polymorphisms) obtained from 1,486 cassava accessions. Six core collections were derived through two optimization strategies based on genetic distances: Average accession-to-nearest-entry and Average entry-to-nearest-entry, along with combinations of phenotypic and genotypic data. The quality of the core collections was evaluated by assessing genetic parameters such as genetic diversity Shannon-Weaver Index, inbreeding (*Fis*), observed (*Ho*), and expected (*Hs*) heterozygosity. While the selection of accessions varied among the six core collections, a seventh collection (consolidated collection) was developed, comprising accessions selected by at least two core collections. Most collections exhibited genetic parameters similar to the complete collection, except for those developed by the Average accession-to-nearest-entry algorithm. However, the variations in the maximum and minimum values of *Ho*, *Hs*, and *Fis* parameters closely resembled the complete collection. The consolidated collection and the collection constructed using genotypic data and the Average entry-to-nearest-entry algorithm (GenEN) retained the highest number of alleles (>97%). Although the differences were not statistically significant (above 5%), the consolidated collection demonstrated a distribution profile and mean trait values most similar to the complete collection, with a few exceptions. The Shannon-Weaver Index of qualitative traits exhibited variations exceeding ±10% when compared to the complete collection. Principal component analysis revealed that the consolidated collection selected cassava accessions with a more uniform dispersion in all four quadrants compared to the other core collections. These findings highlight the development of optimized and valuable core collections for efficient breeding programs and genomic association studies.

## 1 Introduction

Cassava (*Manihot esculenta* Crantz) plays a pivotal role in ensuring global food security as it is a staple food consumed by thousands of people in countries across Africa, Asia, and Latin America (Lebot, 2009). This economically important crop possesses genetic variability. The formal documentation of cassava breeding and *ex situ* conservation of plant genetic resources began in the mid-1930s at the Instituto Agronômico de Campinas in São Paulo (Fukuda et al., 2002). Presently, numerous cassava germplasm collections exist worldwide, with the objective of documenting, evaluating, preserving, and making available the existing genetic diversity of the species for breeding programs. In Brazil, the Empresa Brasileira de Pesquisa e Agropecuária (EMBRAPA) maintains approximately 4,000 *ex situ* conserved cassava accessions in the field and *in vitro* (Hershey, 2017).

The management of these databases is a challenge due to the large number of accessions and the high maintenance cost (Van Hintum et al., 2000). The future of cassava breeding faces potential challenges that could jeopardize its progress, stemming from various factors, including: i) under-representation of the diversity present in certain biomes, ii) the existence of accession duplicates, iii) limited use of germplasm by end users, iv) insufficient regeneration of preserved materials, v) incomplete morpho-agronomic characterization, vi) low investment in the collection and maintenance of resources genetic factors (Díez et al., 2018). Furthermore, the growing demand for the conservation and preservation of genetic materials and the lack of fast methodologies for verifying the existence or not of additional diversity of new germplasms, cause the continuous growth of collections.

Efficient access to genetic variability is crucial for the genetic improvement of successful plants, and large germplasm collections are valuable for preserving genetic diversity (Frankel and Bennett, 1970). However, there is a significant risk that the usefulness and accessibility of these collections will decrease as their size increases (Frankel et al., 1981). Consequently, breeding programs often utilize only a fraction of the available genetic diversity. Germplasm banks play a vital role in storing the genetic variation necessary for continual enhancements in productivity, stress resistance, and nutritional quality through breeding programs (Wang et al., 2017). Nevertheless, cassava germplasm collections consist of thousands of genotypes, including numerous duplicate accessions, with limited characterization and understanding of their potential as parents or for direct use in commercial production systems. This limitation has impeded the utilization of cassava genetic resources to unlock the crop’s full productivity potential and address challenges arising from global climate change.

To address these challenges, various strategies have been proposed to enhance the management of large collections, such as the creation of core collections. Core collections involve selecting representative sets of samples that capture the genetic variability of the entire collection while minimizing redundancy (Brown and Spillane, 1999). Due to their smaller size, core collections can undergo comprehensive phenotyping for key descriptors that define potential applications in species improvement. By characterizing and evaluating a small portion of accessions in detail, core collections can effectively represent the morpho-agronomic and molecular diversity of the complete collection. This approach can encourage researchers and producers to incorporate new germplasms into breeding programs and even directly into production systems (Boczkowska et al., 2016).

Core collections are crucial in maximizing diversity and minimizing duplication within the complete collection, leading to improved management and efficiency in the conservation and utilization of genetic resources for a particular species. In the context of cassava, the formation of core collections was first documented by the International Center for Tropical Agriculture (CIAT) (Hershey, 1994). Bhattacharjee et al. (2012) used 40 morpho-agronomic traits evaluated in two different locations, selecting 428 accessions that captured 90% of the total variation to compose the core collection of the International Institute of Tropical Agriculture (IITA). Similarly, Oliveira et al. (2014) employed 354 single-nucleotide polymorphism (SNP) markers to create core collections with varying numbers of accessions. However, there are limited reports on the formation of cassava core collections that encompass broader genomic coverage and integration of phenotypic and genotypic information.

While phenotypic variation plays a crucial role in practical selection within genetic improvement programs, the establishment of cassava core collections based on a substantial number of morphological and agronomic descriptors evaluated over multiple cultivation years, along with a large set of molecular markers, has not yet been proposed. Therefore, the objectives of this study are as follows: i) develop cassava core collections based on quantitative, qualitative, and molecular data descriptors, both individually and in combination; ii) assess the effectiveness of different selection methods for cassava core collections in retaining maximum genetic diversity, variance, and other genetic parameters compared to the complete collection; and iii) generate a consolidated core collection that represents the highest phenotypic and molecular variability among cassava genotypes.

## 2 Materials and methods

### 2.1 Plant material

A comprehensive evaluation was conducted on a total of 1,486 accessions from the cassava germplasm bank of Embrapa Mandioca e Fruticultura. These accessions originate from various regions in Brazil and some others acquired through exchanges with countries such as Colombia, Venezuela, Nigeria, Mexico and Uganda (**Table S1**). The collection encompasses both local and improved varieties, which have been obtained through breeding techniques such as crossings, mass selection, and identification by producers or research institutions.

The evaluation and characterization of the germplasm bank accessions took place between 2011 and 2021 in three cities in the State of Bahia, Brazil: Cruz das Almas, Laje, and Valença (**Table S2**), according to Fukuda et al. (2010). The climate in this region is classified as type Af according to the Köppen classification, characterized as tropical with an average annual temperature of 24.2°C, approximately 80% humidity, and an average annual precipitation of 1,300 mm. The wettest months typically occur from March to July, while October and January are considered the driest periods. Detailed information regarding soil type, geographic coordinates, and evaluation years for each location can be found in **Table S2**.

### 2.2 Morpho-agronomic descriptors

The characterization of the cassava accessions involved the use of standardized scales for morpho-agronomic descriptors, which encompassed various aspects of the plant including leaf, stem, root, flower, and agronomic traits. The descriptors were categorized into qualitative and quantitative variables specific to the cassava crop, as outlined in **Table S3**. The characterization process followed the guidelines established by Fukuda et al. (2010), Bradbury et al. (1999), and Kawano et al. (1987).

### 2.3 Genotyping of the cassava accessions

DNA extraction from young cassava leaves was carried out using the CTAB method (cetyltrimethylammonium bromide), following the protocol described by Doyle and Doyle (1990) with certain modifications. These modifications included increasing the concentration of 2-mercaptoethanol to 0.4% and incorporating polyvilpyrrolidone (PVP). The quality of the extracted DNA was assessed by running the samples on a 1% agarose gel stained with ethidium bromide (1.0mg/L).

For genotyping, the DNA samples were sent to Cornell University’s Genomic Diversity Facility, where the Genotyping-by-Sequencing (GBS) protocol (Elshire et al., 2011) was employed. Initially, the samples were digested using the restriction enzyme *Ape*KI, a type II restriction endonuclease that recognizes a degenerate sequence of 5 bases (GCWGC, where W represents A or T) with fragment lengths of 100 bp (Hamblin and Rabbi, 2014). After digestion and ligation of the *Ape*KI cleavage fragments with adapters, sequencing was performed in a multiplex system with 192 samples. The Genome Analyzer 2000 genotyping platform (Illumina, Inc., San Diego, CA) was used for the sequencing process. The obtained reads were aligned to the cassava v.6 reference genome (Bredeson et al., 2016) using the BWA software (Li and Durbin, 2009).

A total of 20,023 SNP markers were obtained, and these markers are distributed across the 18 cassava chromosomes. Sequence analysis and quality filtering were performed using Tassel software version 5.2.37 (Bradbury et al., 2007). The filtering steps involved removing markers with a minimum allele frequency (MAF) and a high rate of missing data (Call Rate) below 5% and above 80%, respectively. Any remaining missing data were subsequently imputed using the Beagle 4.1 software (Browning and Browning, 2016).

### 2.4 Data analysis

The accessions in the cassava germplasm bank underwent evaluations in various trials conducted from 2011 to 2021, leading to minor variations in the qualitative descriptors across different classes. To address this variability, we employed the mode as an indicator of the prevailing trend for accessions in terms of qualitative characteristics. The mode signifies the class that exhibited the highest frequency of observations recorded over multiple years.

The quantitative dataset was analyzed using linear mixed models. The dataset included information about the year and location of assessment, referred to as “environments” in this context. A analysis considering all environments for each quantitative descriptor was performed using the following statistical model: y=Zg+Wb+Ti+e , where *y* is the vector of phenotypic observations, *g* represents the genotypic effects considered as random effect $g\sim N\left(0,\sigma_{g}^{2}\right);\;$ *b* is the aligned effects of blocks within trials considered as random $b\sim N\left(0,\sigma_{b}^{2}\right)$ ; *i* represents the effects of the genotype-trial interaction considered as random effect $i\sim N\left(0,\sigma_{i}^{2}\right)$ ; and *e* represents the error effects considered as random effect $e\sim N\left(0,\sigma_{e}^{2}\right)$ . Z, W, and T are the incidence matrices for the corresponding effects. This model was used to estimate the genetic values of the genotypes based on the evaluation of experiments conducted under an incomplete block design across multiple trials. The mixed linear model analyses were performed using the sommer package version 4.1.8 (Covarrubias-Pazaran, 2016) within the R version 4.3.0 environment (R Development Core Team, 2023).

### 2.5 Development of the cassava core collections

Six core collections were generated using different criteria based on distances between the accessions of the complete collection and the core collection (**Table 1**). The core collections were developed by using the stochastic parallel tempering algorithm (Thachuk et al., 2009) in the Core Hunter 3 package version 3.2.2 (Beukelaer and Davenport, 2018) in R version 4.3.0 (R Development Core Team, 2023), selecting 10% of the accessions relative to the size of the complete collection. Two strategies based on genetic distances were employed to optimize the collections: AN (Average accession-to-nearest-entry) and EN (Average entry-to-nearest-entry) as described by Odong et al. (2013). Under the AN criterion, the average distance between the accessions of the complete collection and their closest entry in the core collection is calculated. For the EN criterion, the objective is to maximize the average distance between each entry and its nearest neighboring entry in the complete collection, ensuring that each entry is as distinct as possible from the others. Different datasets were used for each optimization method, including phenotypic data, genotypic data, and a combination of both (phenotypic + genotypic data), each with their respective related distances.

**Table 1:** Summary of strategies used to obtain cassava core collections based on 20,023 SNPs markers and 48 morpho-agronomic descriptors, divided into qualitative (33) and quantitative (15).

Core collection	Entry data	Algoritm	Genetic distance
GenAN	SNPs	AN	Modified Rogers
GenEN		EN	
PhenAN	morpho-agronomic	AN	Gower
PhenEN		AN	
GPmAN	SNPs + morpho-agronomic	AN	Czekanowski (Manhattan) + Gower
GPmEN		EN	

GenAN and GenEN - core collection formed by genotypic data and optimization strategy average accession-to-nearest-entry (AN) and average entry-to-nearest-entry (EN), respectively; PhenAN and PhenEN - Core collection formed by phenotypic data and optimization strategy AN and EN, respectively; GPmAN and GPmEN - Collection formed by morpho-agronomic data + SNPs and optimization strategy AN and EN, respectively.

In the case of collections based solely on phenotypic data, the Gower dissimilarity matrix (Gower, 1971) was employed as a criterion to define the core collections. The Gower matrix allows for the combined analysis of numerical and categorical variables, encompassing both quantitative and qualitative data from the evaluated descriptors. For each variable (*j*), a similarity coefficient ( sj ) within the range of [0,1] is considered. The similarity between elements (*l* and *k*) is then calculated as follows: $d\left(l,k\right)=\left(\frac{\sum_{j=1}^{p+q}1_{j}\left(l,k\right)s_{j}\left(l.k\right)}{\sum_{j=1}^{p+q}1_{j}\left(l,k\right)}\right)$ , where $l_{j}\left(l,k\right)$ is a variable that equals 1 if *l* and *k* can be compared with variable $X_{j}$ .

For collections obtained solely using molecular marker data, the Modified Rogers distance (Wright, 1978) was utilized. The formula for this distance is given as $\left(MR_{ij}=\frac{1}{2L}\sqrt{\sum_{l=1}^{L}\sum_{a=1}^{2}{\left(p_{ila}-p_{jla}\right)}^{2}}\right)$ , where *L* represents the total number of markers, *p_ila_* is the allele frequency of allele *a* of marker l for accession *i*, *p_jla_* is the allele frequency of allele *a* of marker l for accession *j*, and *m_l_* denotes the number of matching alleles for marker l.

To form the pooled core collection, both genotypic and phenotypic data were utilized to generate distance matrices. Gower distance was employed for phenotypic data, while Manhattan distance was applied for genotypic data. The Czekanowski distance (calculated using the Manhattan formula) is given by the equation: $d_{cz}\left(A,B\right)=\frac{1}{2L}\sum_{i=1}^{L}\left|x_{i}-y_{i}\right|$ , where $x_{i}$ and $y_{i}$ are the allele frequencies at locus *i* for individuals A and B, respectively; *L* denotes the number of loci for which $x_{i}$ and $y_{i}$ are available. The implementation of the Czekanowski distance utilized the dartR package version 2.7.2 (Mijangos et al., 2022) in R version 4.3.0 (R Development Core Team, 2023).

### 2.6 Assessing diversity: analysis, comparison, and validation of methods for cassava core collections

The coincidence between the different methods of forming cassava core collections was assessed using the Kappa index (Cohen, 1960). A binary code was employed to represent selected and unselected individuals, where selected individuals were assigned a code of 1 and unselected individuals a code of 0. The coincidence of accessions between collections was then analyzed based on this binary representation.

To evaluate the genetic diversity within core collections, consolidated collection, and complete collection, several parameters were considered. The observed heterozygosity (*Ho*) was calculated using the formula $Ho=1-\;{\;}_{\sum }^{k}{\;}_{\sum }^{i}Pkii/np$ , where Pkii represents the proportion of homozygote i in sample k and np the number of samples. The genetic diversity within the population (*Hs*) was determined using the formula $Hs=ñ/\left(ñ-1\right)\left[1-\sum_{i}\bar{p_{i}^{2}}-Ho/2ñ\right]$ , where $ñ=np/{\;}_{\sum }^{k}1/n_{k}$ and $\bar{p_{i}^{2}}=\sum_{k}P_{ki}^{2}/np$ . The inbreeding coefficient (*Fis*) was calculated as Fis=1−Ho/Hs . These calculations were performed using the hierfstat package version 0.5.11 (Goudet, 2005) in R version 4.3.0 (R Development Core Team, 2023).

The comparison between different core collections, the consolidated collection, and the complete collection for phenotypic data was conducted by analyzing the dispersion of quantitative and qualitative traits. The Shannon-Weaver diversity indices were calculated for each trait in the complete collection and individual collections using the formula $H^{\prime }=-\sum_{i=1}^{n}p_{i}log_{e}p_{i}$ , where pi represents the observed frequency of class *i* for trait *n*, *n* is the number of phenotypic classes. All *H’* indices were normalized and divided by the maximum value ( $log_{e}n$ ) to ensure that the values ranged from 0 to 1, representing monomorphism to maximum phenotypic diversity. For qualitative characteristics, *k* denoted the number of classes or grades of the descriptor, while for quantitative characters, six classes were estimated based on the lower and upper limits observed in the complete collection for each trait (**Table S4**). These analyses were performed using R version 4.3.0 (R Development Core Team, 2023).

The structure of the core and consolidated collections was assessed in comparison to the complete collection using principal component analysis (PCA). Morpho-agronomic data was analyzed using the AMR package version 2.0.0 (Berends et al., 2022), while molecular data underwent PCA using the PCAtools package version 2.12.0 (Blighe and Lun, 2023) in R version 4.3.0 (R Development Core Team, 2023).

## 3 Results

### 3.1 Concordance in genotype selection of core collections using phenotypic, genotypic, and pooled data

**Figure 1:**
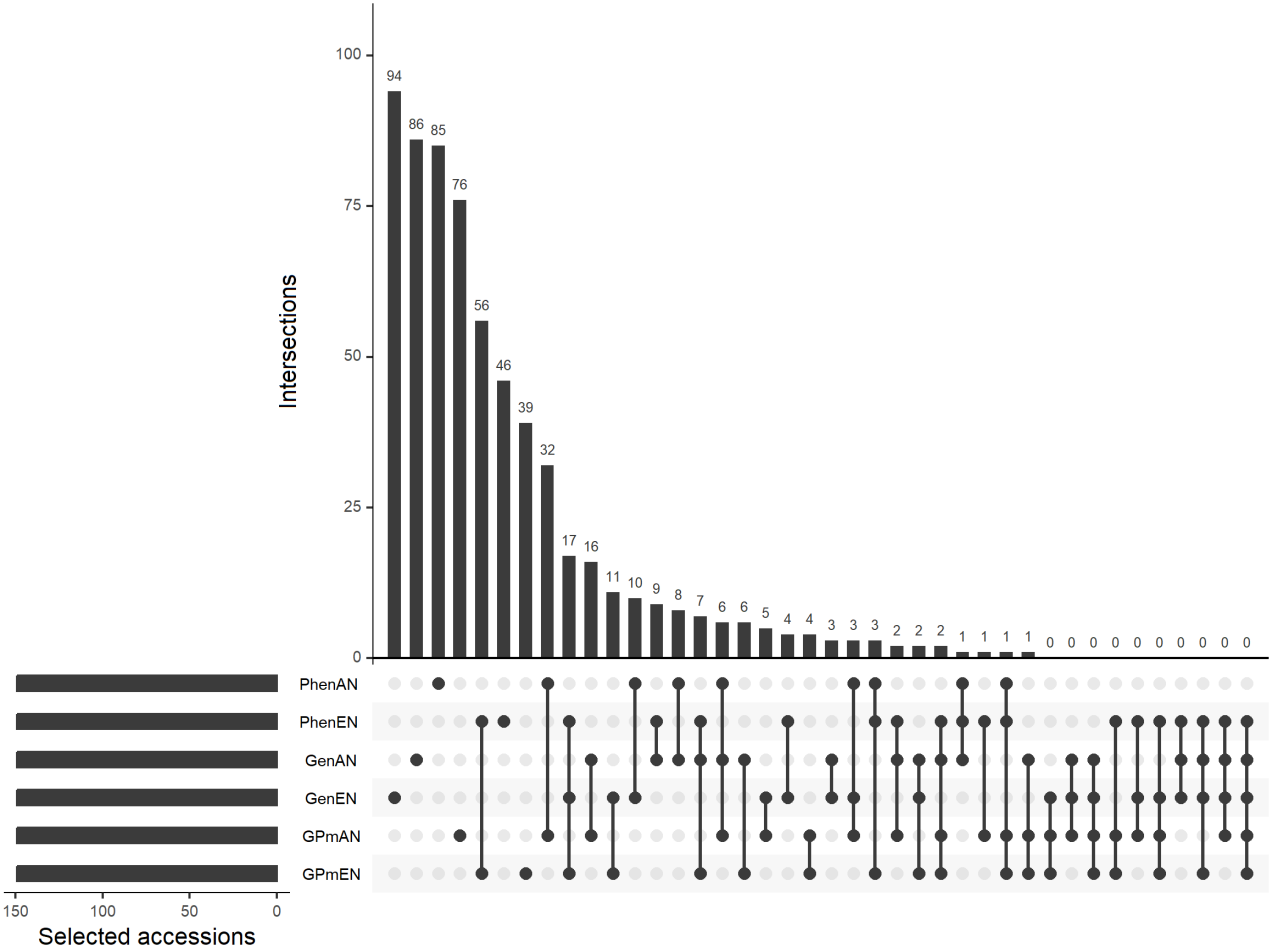
Upset plot of unique and coincidental accessions considering different methodologies for forming core collections: Core collections obtained exclusively with phenotypic data and average entry-to-nearest-entry distance (PhenEN) and average accession-to-nearest-entry optimization algorithm distance (PhenAN); exclusively with genotypic data (GenAN and GenEN); and pooled collections with both phenotypic and genotypic data (GPmAN and GPmEN). Connected bullets represents intersections in core collections formed by different methodologies and bars represents intersection cardinality.

The core collections were created by selecting 10% of the complete collection, resulting in 149 genotypes. Overall, there was a lack of significant overlap in the selected accessions among the core collections. The core collections formed using the EN algorithm, based on phenotypic data (PhenEN), and pooled data (phenotypic and genotypic) (GPmEN) had the fewest exclusive accessions, with 47 and 48, respectively. On the other hand, the remaining core collections had over 50% exclusive accessions, indicating minimal overlap in the selected genotypes (**Figure 1**). Out of the 1486 accessions in the complete collection, 838 were not included in any of the core collections.

The Kappa coefficient was used to assess the concordance in accession selection among the different core collection formation methods, and the results ranged from moderate (0.48) to low (-0.09) (**Table 2**). The highest agreement in genotype selection was observed between the GPmEN and PhenEN collections (0.48) and between GPmAN and PhenAN (0.19). However, most of the coefficients were negative, indicating poorer agreement than would be expected by chance.

**Table 2:** Kappa index considering different methodologies for forming core collections.

Kappa index	GenAN	GenEN	PhenAN	PhenEN	GPmAN	GPmEN
**GenAN**	1	-0.07	0.02	0.05	0.11	0.01
**GenEN**	-0.07	1	-0.04	0.04	-0.05	0.09
**PhenAN**	0.02	-0.04	1	-0.08	0.19	-0.09
**PhenEN**	0.05	0.04	-0.08	1	-0.08	0.48
**GPmAN**	0.11	-0.05	0.19	-0.08	1	-0.09
**GPmEN**	0.01	0.09	-0.09	0.48	-0.09	1

GenAN and GenEN - core collection formed by genotypic data and optimization strategy average accession-to-nearest-entry (AN) and average entry-to-nearest-entry (EN), respectively; PhenAN and PhenEN - Core collection formed by phenotypic data and optimization strategy AN and EN, respectively; GPmAN and GPmEN - Collection formed by morpho-agronomic data + SNPs and optimization strategy AN and EN, respectively.

### 3.2 Establishment of the consolidated core collection

Out of the 1486 cassava accessions, approximately 30% (445 accessions) were exclusively selected by a single type of core collection, while the majority of accessions (~56%) were not included in any core collection (**Table S5**). Since core collections employ different approaches to select important accessions for germplasm maintenance, there is a risk of excluding accessions with alternative alleles/traits from the final core collection. To address this, a seventh core collection called the “consolidated collection” was created, which includes accessions selected by at least two of the previous core collection approaches. The consolidated collection comprised 204 cassava accessions, representing approximately 14% of the complete collection.

Among the core collections, the ones built using the EN algorithm on phenotypic data (PhenEN) and the EN algorithm on grouped data (phenotypic and genotypic) (GPmEN) had the highest number of accessions shared with the consolidated collection, accounting for approximately 50% of the overlaps. The remaining collections had 53 to 69 accessions that were also selected by the consolidated collection. The core collections created using grouped data with the EN and AN algorithms also had a significant number of accessions in common with the consolidated collection (79 accessions) (**Figure S1**). The Kappa coefficient analysis showed positive agreements between the consolidated collection and the core collections. The agreements were of medium magnitude for the PhenEN (0.44) and GPmEN (0.43) collections, and of low magnitude for the GPmAN (0.20), GenAN (0.19), PhenAN (0.1), and GenEN (0.08) collections (**Table S5**).

### 3.3 Parameters analysis: molecular data in the complete collection, core collections, and consolidated collection

To assess the genetic diversity within core and complete collections, genetic parameters were determined using SNP markers (**Table 3**). In general, the majority of collections exhibited comparable genetic parameters to the complete collection, with the exception of those utilizing the AN algorithm, which maintained the same *Ho* value (0.403). Moreover, the variations in the maximum and minimum values for the three parameters (*Ho*, Hs , and Fis ) closely resembled those of the complete collection. Notably, the core collections CCons and GenEN were able to preserve a substantial proportion (>97%) of the total number of alleles present in the complete collections.

**Table 3:** Basic genetic diversity parameters calculated for the core collections formed using different approaches based on 20,023 SNP markers.

Collections	Ho		Hs		Fis		Total number of alleles
	Mean	Range	Mean	Range	Mean	Range	
Complete	0.403	(0.04 – 1.00)	0.301	(0.04 - 0.62)	-0.228	(-1.00/-0.01)	58,672
CCons	0.396	(0.01 – 1.00)	0.299	(0.01 – 0.62)	-0.220	(0.00/-1.00)	56,972
GenAN*	0.403	(0.02 – 1.00)	0.302	(0.02 – 0.62)	-0.226	(0.00/-1.00)	55,976
GenEN	0.390	(0.01 - 1.00)	0.296	(0.01 – 0.62)	-0.213	(0.00/-1.00)	57,338
PhenAN	0.403	(0.01 - 1.00)	0.302	(0.01 – 0.62)	-0.227	(0.00/-1.00)	55,464
PhenEN	0.396	(0.01 - 1.00)	0.298	(0.01 – 0.62)	-0.221	(0.00/-1.00)	56,021
GPmAN	0.403	(0.02 - 1.00)	0.302	(0.02 – 0.62)	-0.226	(-1.00/-0.01)	55,623
GPmEN	0.398	(0.01 - 1.00)	0.299	(0.01 – 0.62)	-0.220	(0.00/-1.00)	56,806

*GenAN and GenEN - core collection formed by genotypic data and optimization strategy average accession-to-nearest-entry (AN) and average entry-to-nearest-entry (EN), respectively; PhenAN and PhenEN - Core collection formed by phenotypic data and optimization strategy AN and EN, respectively; GPmAN and GPmEN - Collection formed by morpho-agronomic data + SNPs and optimization strategy AN and EN, respectively; CCons - consolidated collection that includes accessions selected by at least two of the previous approaches. The genetic diversity parameters assessed were Ho (observed heterozygosity), Hs (genetic diversity within population), and Fis (inbreeding coefficient).

**Figure 2:**
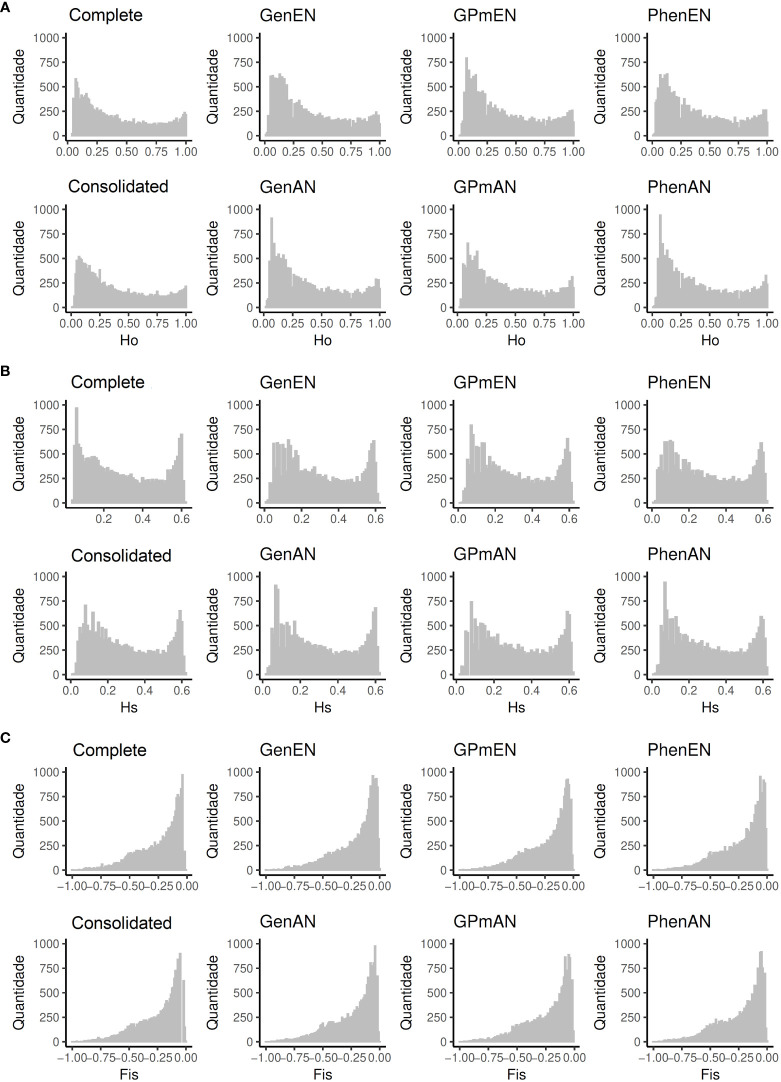
Genetic parameters analyzed for the complete collection (Complete), consolidated collection (CCons), and core collections derived from different data types. GenAN and GenEN - core collection formed by genotypic data and optimization strategy average accession-to-nearest-entry (AN) and average entry-to-nearest-entry (EN), respectively; PhenAN and PhenEN - Core collection formed by phenotypic data and optimization strategy AN and EN, respectively; GPmAN and GPmEN - Collection formed by morpho-agronomic data + SNPs and optimization strategy AN and EN, respectively; CCons - consolidated collection that includes accessions selected by at least two of the previous approaches. The genetic parameters evaluated included: **(A)** observed heterozygosity ( Ho ), **(B)** expected heterozygosity ( Hs ), and **(C)** inbreeding coefficient ( Fis ).

The distribution of genetic parameters in the core collections exhibited patterns that were largely comparable to those observed in the complete collection (**Figure 2**). Notably, prominent similarities were identified between the consolidated collection and the complete collection in terms of the *Ho* parameter. Similarly, the GenAn and PhenAN collections displayed noticeable resemblances to the complete collection in relation to the *Hs* parameter.

### 3.4 Variation in morpho-agronomic descriptors from different core collections

**Figure 3:**
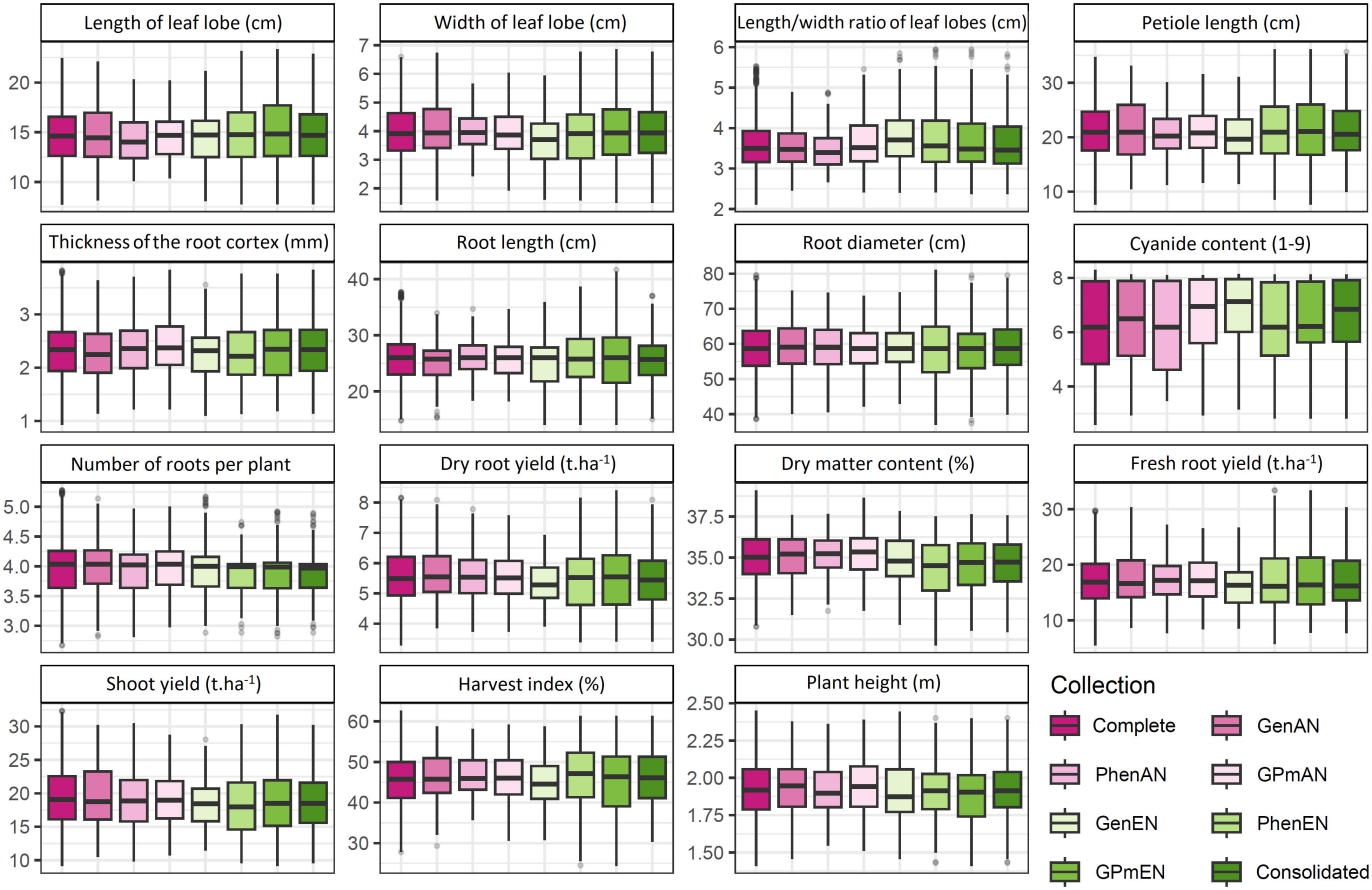
Comparative boxplot analysis of quantitative cassava descriptors in different core collections. GenAN and GenEN - core collection formed by genotypic data and optimization strategy average accession-to-nearest-entry (AN) and average entry-to-nearest-entry (EN), respectively; PhenAN and PhenEN - Core collection formed by phenotypic data and optimization strategy AN and EN, respectively; GPmAN and GPmEN - Collection formed by morpho-agronomic data + SNPs and optimization strategy AN and EN, respectively; CCons - consolidated collection that includes accessions selected by at least two of the previous approaches.

The interquartile ranges of phenotypic traits showed variations among the core collections, although the means of most traits were similar to those of the complete collection. However, some specific traits, such as length and width ratio of leaf lobes, cyanide content, thickness of the root cortex, root diameter, dry matter content, plant height, and harvest index, exhibited slight variations when compared to the complete collection (**Figure 3**). Among the core collections, the consolidated collection displayed a distribution profile and average characteristics that were most similar to the complete collection, with the exception of cyanide content in the roots, number of roots, and harvest index.

The core collections formed based on phenotypic data, whether used alone or in combination with genotypic data, exhibited minimum and maximum values of quantitative traits that were very similar to those of the complete collection. In contrast, collections based solely on genotypic data, such as the GenAN collection, showed greater variation in the mean and range of phenotypic data, particularly for traits related to leaf lobes (e.g., length of leaf lobe, width of leaf lobe, length and width ratio of leaf lobes) and petiole length. Accessions with extreme values or low harvest index were not included in the GenAN core collection.

For the majority of quantitative phenotypic traits, there was no significant difference (>5%) in means and variances between the core collections and the complete collection (**Table S6**). However, some variation was observed in the means of these traits. The GenAN collection showed higher means compared to the complete collection for most traits, except for the length and width ratio of leaf lobes and thickness of the root cortex. Variance was higher than the complete collection (>50%) for certain traits, such as the root diameter, where the GPmEN (125.90) and PhenEN (135.00) collections exhibited considerably higher variances than the complete collection (79.31). The harvest index also showed higher variances than the complete collection (45.95) in the GPmEN (67.00) and PhenEN (66.19) collections. In contrast, the PhenAN collection displayed lower variances than the complete collection for all traits.

**Figure 4:**
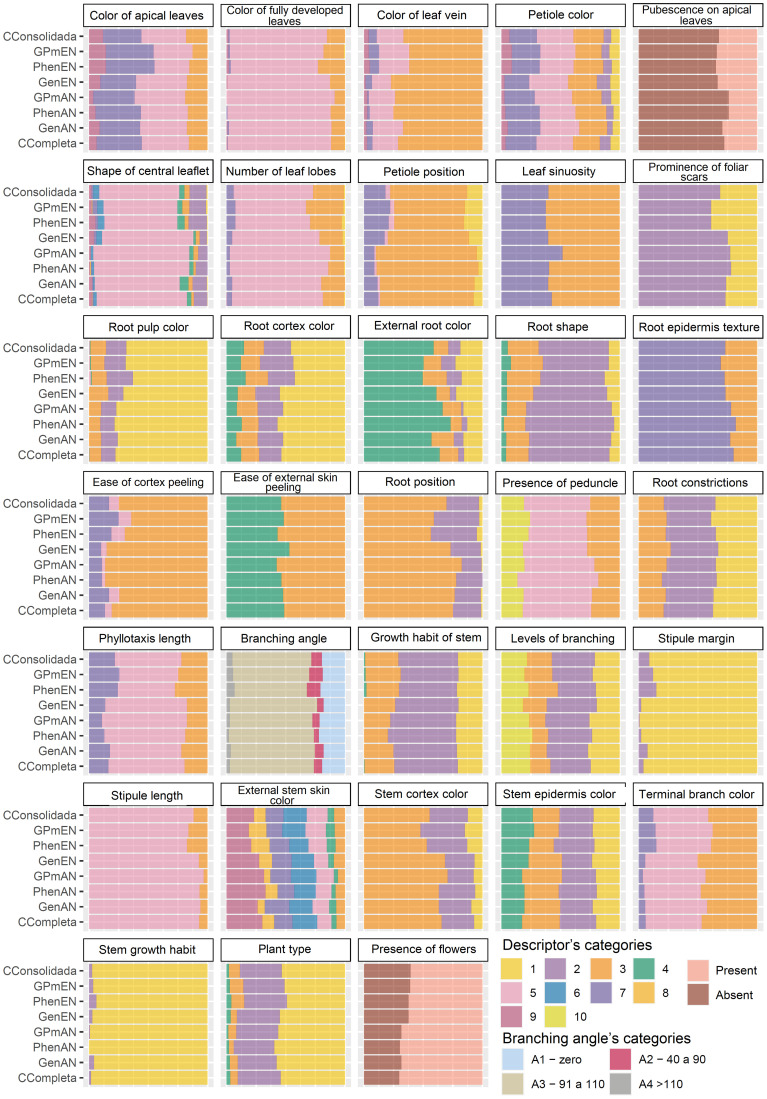
Comparative barplot analysis of different qualitative cassava descriptors across various core collections. GenAN and GenEN - core collection formed by genotypic data and optimization strategy average accession-to-nearest-entry (AN) and average entry-to-nearest-entry (EN), respectively; PhenAN and PhenEN - Core collection formed by phenotypic data and optimization strategy AN and EN, respectively; GPmAN and GPmEN - Collection formed by morpho-agronomic data + SNPs and optimization strategy AN and EN, respectively; CCons - consolidated collection that includes accessions selected by at least two of the previous approaches.

The qualitative data were analyzed based on the efficiency of the core collections in encompassing all classes of each evaluated trait (**Figure 4**). The core collections that showed a better balance in representing the classes were CCons, GPmEN, and PhenEN, especially for the traits of color of leaf vein, number of lobes, petiole position, root pulp color, root position, stipule margin, growth habit of stem, branching angle, and external stem skin color.

Some characteristics were not well represented in all core collections, possibly because some of the classes are rare occurrences, being seldom observed in the field. For example, only 28 cassava accessions (~1.88% of the complete collection) exhibit a zig-zag stem growth habit. Therefore, any variation in the method of forming the core collection can alter this frequency, as seen in the PhenAN collection, which was represented only by the straight growth habit. Other significant variations in the representativeness of the core collections based on qualitative data were identified for the stipule margin and root position traits, also due to the low frequency of certain classes in the complete collection.

### 3.5 Analysis of the phenotypic diversity of core collections

The quality assessment of core collections was conducted using the Shannon-Weaver Index (ISW). Comparisons were made between the core collections and the complete collection, with variations greater than ±10% of the ISW considered significant for quantitative phenotypic traits (**Table 4**). Among the core collections, only the thickness of the root cortex showed a significant impact on the ISW, reaching 0.00 in the GenAN, PhenAN, GPmAN, and CCons collections, while being higher than the complete collection in the GenEN, PhenEN, and GPmEN collections. For other traits, the GenAN collection exhibited the smallest difference in ISW compared to the complete collection, while the PhenAN collection had the highest number of traits with lower ISW than the complete collection (length of leaf lobe, length and width ratio of leaf lobes, petiole length, root length, and harvest index). On the other hand, the PhenEN and GPmEN collections had a greater number of traits with higher ISW than the complete collection (length and width ratio of leaf lobes, petiole length, thickness of the root cortex, root length, root diameter, and harvest index). The consolidated collection showed minor differences in ISW for most traits, except for the length and width ratio of leaf lobes, root length and diameter, dry root yield, and harvest index, where the ISW differences exceeded 5%.

**Table 4:** Shannon-Weaver indices obtained based on 15 quantitative descriptors evaluated in cassava accessions for the development of different core collections.

Trait	Collection							
	Complete	GenAN	GenEN	PhenAN	PhenEN	GPmAN	GPmEN	CCons
Length of leaf lobe	0.80	0.80	0.76	0.68	0.87	0.71	0.88	0.80
Width of leaf lobe	0.82	0.84	0.82	0.76	0.86	0.77	0.90	0.85
Length and width ratio leaf lobes	0.50	0.49	0.59	0.38	0.58	0.52	0.57	0.55
Petiole length	0.70	0.75	0.65	0.62	0.79	0.64	0.80	0.72
Thickness of the root cortex	0.01	0.00	0.02	0.00	0.02	0.00	0.02	0.00
Root length	0.46	0.49	0.50	0.34	0.52	0.41	0.52	0.51
Root diameter	0.59	0.53	0.53	0.56	0.72	0.55	0.68	0.64
Cyanide content	0.86	0.86	0.79	0.85	0.86	0.83	0.83	0.84
Number of roots per plant	0.68	0.72	0.71	0.66	0.73	0.64	0.74	0.71
Dry root yield	0.73	0.68	0.67	0.70	0.75	0.68	0.76	0.69
Dry matter content	0.72	0.68	0.70	0.64	0.75	0.69	0.78	0.73
Fresh root yield	0.77	0.77	0.72	0.73	0.83	0.74	0.82	0.79
Shoot yield	0.79	0.78	0.77	0.72	0.82	0.75	0.81	0.77
Harvest index	0.77	0.77	0.78	0.67	0.86	0.74	0.87	0.83
Plant height	0.71	0.72	0.70	0.65	0.76	0.73	0.78	0.74

Complete collection of cassava germplasm (Complete), GenAN and GenEN - core collection formed by genotypic data and optimization strategy average accession-to-nearest-entry (AN) and average entry-to-nearest-entry (EN), respectively; PhenAN and PhenEN - Core collection formed by phenotypic data and optimization strategy AN and EN, respectively; GPmAN and GPmEN - Collection formed by morpho-agronomic data + SNPs and optimization strategy AN and EN, respectively; CCons - consolidated collection that includes accessions selected by at least two of the previous approaches.

The ISW for qualitative traits exhibited variations greater than ±10% when compared to the complete collection, particularly in the GenEN, PhenAN, PhenEN, GPmAN, GPmEN, and CCons collections (**Table 5**). Similar to the quantitative traits, the GenAN collection demonstrated the lowest ISW variation for qualitative traits, except for growth habit of stem, stipule margin, external root color, and ease of cortex peeling, which exhibited higher ISW compared to the complete collection. However, stipule length showed a reduction in ISW. In the core collections PhenEN, GPmEN, and CCons, there was a trend towards an increase in ISW compared to the complete collection for most traits (color of fully developed leaf, color of leaf vein, shape of the central leaflet, number of lobes, position of the petiole, prominence of foliar scars, stipule length, terminal branch color, phyllotaxis length, branching angle, stem cortex color, stem growth habit, stipule margin, root pulp color, external root color, ease of external skin peeling, root shape, root epidermis texture, and presence of flowers). On the other hand, the PhenAN and GPmAN collections showed a tendency to reduce the ISW, especially for the color of leaf vein, shape of central leaflet, number of lobes, position of the petiole, stem growth habit, stipule margin, plant type, external root color, root cortex prominence, root shape, root position, and presence of root peduncle.

**Table 5:** Shannon-Weaver indices obtained based on 33 qualitative descriptors of leaf, stem, root and flower, evaluated in cassava accessions for the development of different core collections.

Trait		Collection							
		Complete	GenAN	GenEN	PhenAN	PhenEN	GPmAN	GPmEN	CCons
Leaf	Color of apical leaves	0.87	0.90	0.90	0.86	0.93	0.84	0.92	0.93
	Color of fully developed leaves	0.26	0.29	0.26	0.25	0.43	0.18	0.40	0.34
	Color of leaf vein	0.58	0.61	0.51	0.51	0.72	0.51	0.71	0.66
	Shape of central leaflet	0.42	0.47	0.43	0.35	0.59	0.34	0.60	0.52
	Petiole color	0.89	0.86	0.88	0.84	0.91	0.87	0.92	0.89
	Pubescence on apical leaves	0.85	0.88	0.92	0.80	0.93	0.80	0.93	0.91
	Number of leaf lobes	0.43	0.42	0.48	0.34	0.58	0.33	0.57	0.52
	Petiole position	0.50	0.50	0.66	0.39	0.77	0.40	0.75	0.71
	Leaf sinuosity	0.98	0.97	0.97	0.97	0.96	1.00	0.96	0.97
	Prominence of foliar scars	0.84	0.83	0.81	0.76	0.96	0.78	0.96	0.90
Stem	Stipule length	0.37	0.33	0.38	0.36	0.67	0.21	0.64	0.52
	Terminal branch color	0.79	0.78	0.79	0.77	0.92	0.74	0.91	0.96
	Phyllotaxis length	0.82	0.86	0.76	0.76	0.96	0.71	0.95	0.90
	Branching angle	0.61	0.62	0.57	0.56	0.76	0.61	0.72	0.69
	External stem skin color	0.92	0.93	0.94	0.91	0.97	0.89	0.97	0.95
	Stem cortex color	0.59	0.63	0.57	0.59	0.70	0.56	0.72	0.69
	Stem epidermis color	0.98	0.99	1.00	0.98	0.98	0.98	0.99	1.00
	Stem growth habit	0.12	0.24	0.18	0.00	0.33	0.06	0.21	0.17
	Stipule margin	0.24	0.38	0.14	0.14	0.60	0.06	0.53	0.43
	Plant type	0.71	0.69	0.70	0.64	0.78	0.67	0.76	0.72
	Growth habit of stem	0.75	0.73	0.73	0.70	0.80	0.72	0.79	0.77
	Levels of branching	0.98	0.97	0.95	0.96	0.99	0.96	0.98	0.98
Root	Root pulp color	0.43	0.45	0.50	0.42	0.61	0.43	0.55	0.55
	Root cortex color	0.86	0.85	0.84	0.83	0.95	0.86	0.92	0.92
	External root color	0.73	0.82	0.75	0.62	0.89	0.67	0.88	0.81
	Ease of cortex peeling	0.55	0.67	0.47	0.42	0.74	0.42	0.79	0.66
	Ease of external skin peeling	1.00	1.00	1.00	1.00	0.99	0.98	1.00	1.00
	Root shape	0.66	0.65	0.73	0.54	0.79	0.62	0.80	0.75
	Root epidermis texture	0.72	0.78	0.84	0.68	0.85	0.76	0.89	0.84
	Root position (RP)	0.55	0.54	0.58	0.48	0.76	0.45	0.71	0.65
	Presence of peduncle	0.88	0.88	0.91	0.76	0.95	0.87	0.96	0.90
	Root constrictions	0.97	0.98	0.99	0.95	0.97	0.98	0.98	0.89
Flower	Presence of flowers	0.88	0.90	0.96	0.88	0.96	0.90	0.96	0.97

GenAN and GenEN - core collection formed by genotypic data and optimization strategy average accession-to-nearest-entry (AN) and average entry-to-nearest-entry (EN), respectively; PhenAN and PhenEN - Core collection formed by phenotypic data and optimization strategy AN and EN, respectively; GPmAN and GPmEN - Collection formed by morpho-agronomic data + SNPs and optimization strategy AN and EN, respectively; CCons - consolidated collection that includes accessions selected by at least two of the previous approaches.

### 3.6 Validation of core collections

**Figure 5:**
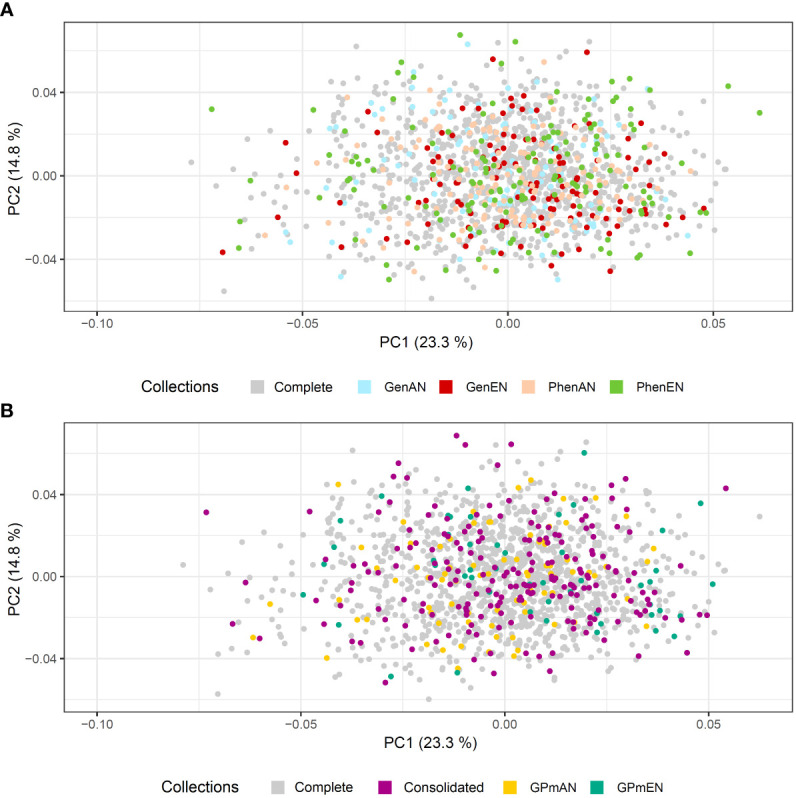
Principal component analysis (PCA) of the phenotypic data of 1,486 cassava accessions with dispersion of different core collections. **(A)** Complete collection (CComplete) and GenAN and GenEN - core collection formed by genotypic data and optimization strategy average accession-to-nearest-entry (AN) and average entry-to-nearest-entry (EN), respectively; PhenAN and PhenEN - Core collection formed by phenotypic data and optimization strategy AN and EN, respectively. **(B)** Complete collection (CComplete), CCons - consolidated collection that includes accessions selected by at least two of the previous approaches, and GPmAN and GPmEN - Collection formed by morpho-agronomic data + SNPs and optimization strategy AN and EN, respectively.

**Figure 6:**
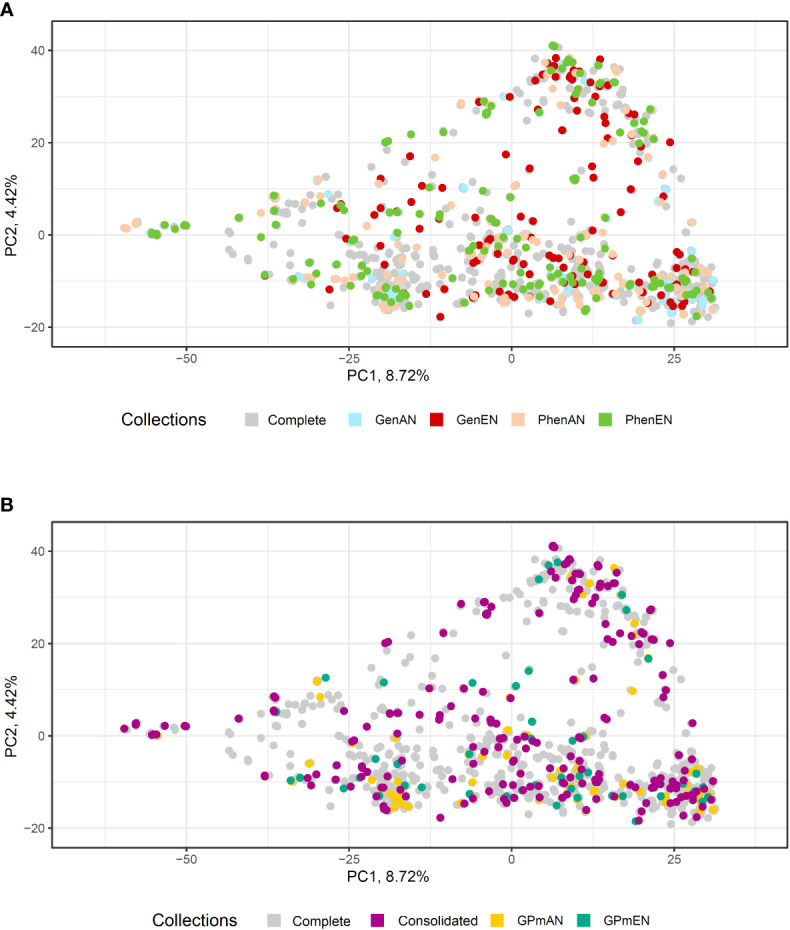
Principal component analysis (PCA) based on 20,023 single-nucleotide polymorphisms (SNP) of 1486 cassava accessions with dispersion of different core collections. **(A)** Complete collection (CComplete) and GenAN and GenEN - core collection formed by genotypic data and optimization strategy average accession-to-nearest-entry (AN) and average entry-to-nearest-entry (EN), respectively; PhenAN and PhenEN - Core collection formed by phenotypic data and optimization strategy AN and EN, respectively. **(B)** Complete collection (CComplete), CCons - consolidated collection that includes accessions selected by at least two of the previous approaches, and GPmAN and GPmEN - Collection formed by morpho-agronomic data + SNPs and optimization strategy AN and EN, respectively.

Principal Component Analysis (PCA) was utilized to evaluate the representation of diversity in the core collections based on phenotypic and molecular data (**Figures 5**, **6**, respectively). The first and second principal components accounted for over 38% of the phenotypic variation in cassava accessions, indicating a good representation of phenotypic diversity (**Figure 5**). Overall, the selected accessions in the different core collections were well distributed across the quadrants of the phenotypic data PCA. However, the GPmEN collection exhibited a higher number of cassava accessions positioned at the extremes of the phenotypic data PCA quadrants, while the consolidated collection demonstrated a slightly more uniform dispersion of cassava accessions across all four quadrants compared to the other collections.

In the PCA analysis of SNPs, the first two principal components accounted for a smaller percentage of the molecular variation in the data (8.72% and 4.42%, respectively) compared to the phenotypic data (**Figure 6**). Despite this, similar to the phenotypic data PCA, all core collections were well represented in the molecular data PCA. The consolidated collection and GPmEN exhibited accessions distributed across all quadrants and had a more representative distribution compared to the complete collection.

## 4 Discussion

### 4.1 Convergence of selection and diversity in phenotypic and molecular data of core collections

A core collection is a subset of accessions derived from larger germplasm collections with the goal of representing the maximum possible diversity of the original collection (Frankel & Brown, 1984). It is generally recommended to develop core collections that have at least 10% of the size and 70% of the genetic diversity of the original collection (Brown, 1989). Following this recommendation, several core collections of cassava have been constructed using phenotypic and genotypic data alone or in combination, along with a consolidated collection that includes accessions selected by at least two core collections. However, the selection of cassava accessions based on phenotypic and genotypic data did not show high agreement. This lack of correlation between morphological and molecular data has also been observed in potato populations (*Solanum tuberosum* L.) (Berdugo-Cely et al., 2017). The discrepancy can be attributed to the selection pressures that populations undergo, as molecular markers are generally not subject to natural selection, while phenotypic traits are influenced by selection pressures and environmental factors. Another explanation for the low agreement in selection is the weak association between the genomic regions accessed by SNPs and the evaluated phenotypic traits (Oliveira et al., 2012).

Several core collections have been developed based on phenotypic data alone (Upadhyaya and Ortiz, 2001; Upadhyaya et al., 2009; Mahmoodi et al., 2019). Although phenotypic data is directly related to agronomic and yield attributes, it can be influenced by environmental factors, experimental errors, and genotype × environment interactions. Therefore, it is recommended to construct core collections that incorporate both phenotypic and genotypic data to ensure maximum representativeness of the original collection for a wide range of data types and characteristics (Kumar et al., 2016; Xue et al., 2021), without losing important alleles for conservation and improvement purposes.

Due to the low agreement in the selection of cassava accessions among different core collections and the risk of excluding accessions with important phenotypic or molecular characteristics, a consolidated core collection was created by including accessions selected by at least two methodological approaches (EN and AN) and different types of collections (Gen, Phen, and GPm). This slightly increased the number of selected clones (from 10% to ~14% of the complete collection), which is still manageable within the scope of genetic resources and species breeding programs.

Overall, the cassava core collections effectively retained a high number of SNP alleles from the complete collection, surpassing 94.5%. Notably, the consolidated and GenEN collections exhibited the highest allelic richness, retaining 97.1% and 97.73% of the alleles, respectively. This preservation of allelic richness in core collections holds significant importance for future studies on genomic associations, especially for traits controlled by rare alleles. Furthermore, the allelic richness retained in cassava core collections compares favorably to other species such as maize (93% - Todorovska et al., 2005) and tomato (92% - Martins et al., 2015), indicating promising results.

While minimal changes were observed in the analyzed genetic parameters, core collections constructed based on phenotypic and genotypic information separately exhibited greater deviations in diversity values and genetic parameters (*Ho*, *Hs*, and *Fis*) compared to the complete and consolidated collections. Methodologically, the collections obtained through the AN algorithm demonstrated *Ho*, *Hs*, and *Fis* values more similar to the complete collection, likely due to the algorithm’s aim of achieving a similar representation of the complete collection’s characteristics in the core collection. In general, the variation of *Ho* (0.39 to 0.40) and *Hs* (0.29 to 0.30) observed in core collections for biallelic markers closely aligns with other cassava germplasms, such as the global cassava collection from IITA, which exhibited *Ho* values ranging from 0.33 to 0.39 and *Hs* ranging from 0.31 to 0.34 (Ferguson et al., 2019).

The distribution and representativeness of the core collections demonstrated relative similarity to the complete collection, considering the mean and variance estimates of the quantitative phenotypic data. However, the distribution profile of the means and variances of the phenotypic data more closely resembled that of the complete collection. Similar findings of few significant differences between the complete collection and core collections were reported in studies on maize landraces (Li et al., 2005) and rice (*Oryza sativa* L.) (Yan et al., 2009). These results highlight the high potential of these core collections to represent the complete collection without significant losses in genetic variability.

In specific cases, an increase in the variance of certain traits was observed, likely due to the removal of accessions that made redundant contributions to phenotypic diversity, with phenotypic values close to the sample mean. For instance, the mean root diameter variance in the consolidated collections, GPmEN, and PhenEN increased by 31%, 49%, and 62%, respectively, compared to the complete collection. However, this increase in variance within core collections is a desirable attribute as it allows for a greater representation of the complete collection while minimizing redundancy (Hu et al., 2000). This is important for obtaining manageable collections that can be practically used in discovering new characteristics and incorporating them into the species improvement efforts.

The ISW was also utilized to assess the efficiency of core collections in representing cassava germplasm diversity. Overall, variations greater than ±10% of the ISW compared to the complete collection were observed for only a few quantitative phenotypic traits. The characteristics that were most affected by the ISW reaching 0.00 in the collections obtained with the AN algorithm were those with the greatest imbalance in their distribution, such as the thickness of the root cortex. On the other hand, the collections obtained with the EN algorithm exhibited higher ISW values than the complete collection. Similar results were identified in the analysis of qualitative phenotypic data, where the collections based on the EN algorithm and the consolidated collection showed higher ISW compared to the complete collection for most traits. However, similar to other species with clonal propagation such as yam (Beukelaer and Davenport, 2018; Girma et al., 2018), cassava core collections maintained or even increased diversity based on ISW among the core collections.

### 4.2 Optimizing diversity and representation in core collections: strategies for effective utilization of germplasm resources

The construction of core collections involves various methodological considerations that can impact the representativeness of genetic diversity. In this study, two criteria described by Odong et al. (2013) were employed to create the core collections, each serving a distinct purpose. The Type 1 collection aimed to maximize the genetic diversity of the complete collection by encompassing all original diversity. This type of collection ensured a more balanced representation of phenotypic characteristics, including those with both low and high frequencies. The collections formed using the AN algorithm, which minimized the average distance between accessions in the complete dataset and the closest selected accession in the core collection, maintained similar levels of heterozygosity, genetic diversity, and inbreeding coefficients compared to the complete collection.

The second collection in this study, known as type 3 aimed to represent the distribution of accessions in the complete collection. Its objective was to ensure that the selected proportion of the complete collection reflects the numerical contributions of different categories in the core collection. The EN algorithm was used to form these collections, which selected accessions that were well-distributed, particularly at the extremes of the different quadrants of the PCA. This approach provided a better representation of the entire collection, resulting in more diverse collections where each selected individual was sufficiently different from others. As a result, subsets with low redundancy (Odong et al., 2013) and high representativeness of the descriptors used to form the collections were obtained. This increased sample diversity was evident when considering the ISW.

The consolidated collection was developed to address the issue of low coincidence in the selection of accessions among the core collections. It served as an alternative to better represent the cassava accessions among the six collections developed based on different types of data. The consolidated collection proved to be efficient not only in overcoming the low coincidence but also in improving allele retention. It resulted in less difference in genetic parameters among the collections and maintained maximum diversity in the ISW for all traits. Furthermore, it better represented the phenotypic and genotypic classes of the complete collection in the PCA.

### 4.3 Validating the effectiveness of core collections: enhancing representation and retention of genetic diversity

The distribution of selected accessions in the core and consolidated collections of cassava exhibited a remarkable level of representativeness when compared to the complete collection, as evidenced by the PCA analysis conducted on both phenotypic and molecular data. Despite the presence of population structure in both data sets, the cassava accessions were well dispersed across different quadrants of the PCA, with notable emphasis on the GPmEN and consolidated collections. This resulted in the selection of cassava accessions with minimal redundancy within the core collections. Similar studies conducted on other species, such as *Lagenaria siceraria*, have also demonstrated that PCA analysis of core collections, utilizing various phenotypic data types, accurately represents the complete collection and preserves the geographic distribution of accessions (Wang et al., 2021).

It is important to acknowledge that there is no universally applicable ratio or fixed size for all core collections, as the research requirements vary among different species. Nevertheless, the consolidated collection outlined in this study, which comprises approximately 14% of the complete collection, exhibits an appropriate sample ratio considering the extensive breadth and complexity of cassava genetic resources. This consolidated collection serves as a valuable and comprehensive reference, forming a solid basis for the utilization of cassava germplasm resources in future breeding programs.

### 4.4 Cassava core collections for conservation, characterization and use of cassava genetic resources

The conservation of cassava genetic resources is crucial for research purposes and the discovery of genes with agronomic significance to be used in cassava breeding programs. However, Guo et al. (2014) highlighted the challenge of maintaining and utilizing the diversity of accessions in a germplasm bank. The entire process of conservation and characterization is labor-intensive, time-consuming, and requires substantial financial resources. In this context, the technological advancements developed in this study offer a relevant alternative for reducing costs associated with the conservation and characterization stages of cassava germplasm.

The main objective of developing the core collection, in addition to reducing the size of the set and maintaining genetic representativeness, is to define conservation priorities, prioritize and allocate efforts for characterizations and evaluations, facilitate access, and enhance knowledge of the available genetic structure in germplasm banks. The consolidated core collection will facilitate the handling of a more focused and detailed morphological and agronomic variability, enabling comprehensive characterization studies. These measures aim to optimize the conservation and utilization of cassava germplasm while ensuring the preservation of currently available genetic resources. Moreover, this core collection will be given priority for *in vitro* conservation, ensuring protection against environmental degradation and facilitating efficient exchange of the collection. It is important to note that genetic collections should be dynamic and periodically reviewed to incorporate additional accessions. This ensures that the most valuable genotypes are preserved and characterized, serving the purpose of conservation and species improvement.

## 5 Conclusion

This study highlights the possibility of using diverse methodological approaches and data types to construct core collections for cassava, effectively preserving the diversity and genetic parameters of the complete collection. However, the low overlap in the selection of accessions among different core collection formation algorithms necessitated the creation of an alternative collection called the consolidated collection. This collection incorporated cassava accessions selected by at least two different algorithms, combining phenotypic and genotypic data.

The consolidated collection demonstrated less variation in the analyzed genetic parameters compared to the complete collection. It retained over 97% of the allelic richness observed in the complete collection, even with the inclusion of accessions selected based on different types of information. Additionally, the consolidated collection exhibited similar data dispersion and representation of classes in both quantitative and qualitative characteristics when compared to the complete collection. Despite representing a larger percentage of the complete collection than initially planned (approximately 14%), the consolidated collection remains manageable in size, allowing for efficient characterization and utilization of the germplasm. Overall, the formation of the consolidated collection addresses the challenge of low coincidence in accession selection and provides a robust and representative resource for further research and breeding programs in cassava.

## Data availability statement

The original contributions presented in the study are publicly available. This data can be found here: https://doi.org/10.6084/m9.figshare.23818875.v1.

## Author contributions

LD, EO designed the study. CS performed the field evaluations. CS, LD, and CC analyzed the data. CS, and CC wrote the paper. All authors contributed to the article and approved the submitted version.
