# Supplementary material for: Development of cassava core collections based on morphological and agronomic traits and SNPS markers
Source: Front Plant Sci. 2023 Sep 6;14:1250205. doi: 10.3389/fpls.2023.1250205 (PMC10511765; doi:10.3389/fpls.2023.1250205)
Supplement: Supplementary file 1 [file DataSheet_1.zip › Table 2 (75).docx]

**Supplement**

**Table S2**. Geographical location and field characterization of the trials implemented to evaluate the cassava germplasm.

| Year | Experimental design | Location | City | Altitude | Coordenates | Soil type^1^ |
| --- | --- | --- | --- | --- | --- | --- |
| 2011 | Completely randomized | Embrapa | Cruz das Almas | 215 | 12°40'36.7"S, 39°05'08.0"W | LA |
|  | Completely randomized | Embrapa | Cruz das Almas | 210 | 12°40'32.6"S, 39°05'13.7"W | LA |
| 2012 | Augmented Block | Embrapa | Cruz das Almas | 215 | 12°40'36.7"S, 39°05'08.0"W | LA |
|  | Augmented Block | Embrapa | Cruz das Almas | 210 | 12°40'32.6"S, 39°05'13.7"W | LA |
|  | Augmented Block | Embrapa | Cruz das Almas | 200 | 12°40'47.4"S, 39°05'00.2"W | LA |
| 2013 | Completely randomized | Coopamido | Laje | 180 | 13°06'38.4"S, 39°16'20.4"W | LV |
|  | Completely randomized | UFRB | Cruz das Almas | 210 | 12°39'25.9"S, 39°04'58.8"W | LA |
|  | Completely randomized | Embrapa | Cruz das Almas | 210 | 12°40'32.6"S, 39°05'13.7"W | LA |
|  | Completely randomized | Embrapa | Cruz das Almas | 217 | 12°40'22.8"S, 39°05'06.1"W | LA |
| 2014 | Augmented Block | Embrapa | Cruz das Almas | 216 | 12°40'22.8"S, 39°05'01.5"W | LA |
|  | Augmented Block | Embrapa | Cruz das Almas | 216 | 12°40'22.8"S, 39°05'01.5"W | LA |
|  | Augmented Block | Coopamido | Laje | 180 | 13°06'39.6"S, 39°16'17.6"W | LA |
|  | Augmented Block | Coopamido | Laje | 175 | 13°06'35.6"S, 39°16'19.3"W | LA |
|  | Augmented Block | Embrapa | Cruz das Almas | 216 | 12°40'22.8"S, 39°05'01.5"W | LA |
|  | Completely randomized | Coopamido | Laje | 180 | 13°06'39.6"S, 39°16'17.6"W | LA |
| 2015 | Augmented Block | Embrapa | Cruz das Almas | 216 | 12°40'22.8"S, 39°05'01.5"W | LA |
|  | Augmented Block | Bahiamido | Laje | 196 | 13°06'38.5"S, 39°16'49.0"W | LA |
|  | Augmented Block | Bahiamido | Valença | 40 | 13°15'33.5"S, 39°14'12.8"W | LVA |
|  | Augmented Block | UFRB | Cruz das Almas | 210 | 12°39'16.4"S, 39°04'53.4"W | LA |
|  | Augmented Block | Bahiamido | Laje | 296 | 13°06'38.5"S, 39°16'49.0"W | LA |
|  | Augmented Block | Embrapa | Cruz das Almas | 216 | 12°40'19.5"S, 39°05'02.5"W | LA |
| 2016 | Augmented Block | Embrapa | Cruz das Almas | 216 | 12°40'19.5"S, 39°05'02.5"W | LA |
|  | Augmented Block | Embrapa | Cruz das Almas | 216 | 12°40'19.5"S, 39°05'02.5"W | LA |
|  | Augmented Block | Embrapa | Cruz das Almas | 216 | 12°40'19.5"S, 39°05'02.5"W | LA |
| 2017 | Augmented Block | Embrapa | Cruz das Almas | 216 | 12°40'19.5"S, 39°05'02.5"W | LA |
| 2018 | Completely randomized | UFRB | Cruz das Almas | 223 | 12°39'51.4"S, 39°04'15.7"W | LA |
| 2019 | Completely randomized | UFRB | Cruz das Almas | 223 | 12°39'43.5"S, 39°04'12.0"W | LA |
|  | Completely randomized | UFRB | Cruz das Almas | 223 | 12°39'43.5"S, 39°04'12.0"W | LA |
|  | Completely randomized | UFRB | Cruz das Almas | 223 | 12°39'43.5"S, 39°04'12.0"W | LA |
| 2020 | Completely randomized | UFRB | Cruz das Almas | 223 | 12°39'49.2"S, 39°03'58.1"W | LA |
|  | Completely randomized | UFRB | Cruz das Almas | 225 | 12°39'49.2"S, 39°03'58.1"W | LA |
| 2021 | Augmented Block | UFRB | Cruz das Almas | 225 | 12°39'49.2"S, 39°03'58.1"W | LA |

^1^LA – yellow latosol; LV – red latosol; LVA – yellow-red latosol
