# Supplementary material for: Development of cassava core collections based on morphological and agronomic traits and SNPS markers
Source: Front Plant Sci. 2023 Sep 6;14:1250205. doi: 10.3389/fpls.2023.1250205 (PMC10511765; doi:10.3389/fpls.2023.1250205)
Supplement: Supplementary file 1 [file DataSheet_1.zip › Table 3 (41).DOCX]

**Supplement**

**Table S3.** Qualitative and quantitative descriptors evaluated for the formation of phenotypic and genotypic core collections.

| Plant organ | Qualitative Descriptor | Quantitative Descriptor |
| --- | --- | --- |
| Leaf | Color of apical leaves | Length of leaf lobe (cm) |
|  | Color of fully developed leaves | Width of leaf lobe (cm) |
|  | Color of leaf vein | Length and width ratio of leaf lobes |
|  | Shape of central leaflet | Petiole length (cm) |
|  | Petiole color |  |
|  | Pubescence on apical leaves |  |
|  | Number of leaf lobes |  |
|  | Petiole position |  |
|  | Leaf sinuosity |  |
| Stem | Prominence of foliar scars | Plant height (cm) |
|  | Stipule length | Dry matter contente (Kawano et al., 1987) |
|  | Terminal branch color | Shoot yield (t.ha^1^) |
|  | Phyllotaxis length | Harvest index (%) |
|  | Branching angle |  |
|  | External stem skin color |  |
|  | Stem cortex color |  |
|  | Stem epidermis color |  |
|  | Stem growth habit |  |
|  | Stipule margin |  |
|  | Plant type |  |
|  | Growth habit of stem |  |
|  | Levels of branching |  |
| Root | Root pulp color | Thickness of the root córtex (mm) |
|  | Root cortex color | Root diameter (cm) |
|  | External root color | Root length (cm) |
|  | Ease of cortex peeling | Cyanide content (Bradbury et al., 1999) |
|  | Ease of external skin peeling | Number of roots per plant |
|  | Root shape | Fresh root yield (t.ha^1^) |
|  | Root epidermis texture | Dry root yield (t.ha^1^) |
|  | Root position |  |
|  | Presence of peduncle |  |
|  | Root constrictions |  |
| Flower | Presence of flowers |  |
