# Supplementary material for: Development of cassava core collections based on morphological and agronomic traits and SNPS markers
Source: Front Plant Sci. 2023 Sep 6;14:1250205. doi: 10.3389/fpls.2023.1250205 (PMC10511765; doi:10.3389/fpls.2023.1250205)
Supplement: Supplementary file 1 [file DataSheet_1.zip › Table 4 (24).DOCX]

**Supplement**

**Table S4**. Estimation of lower and upper limits of phenotypic classes to be used for estimating the Shannon-Weaver diversity index for quantitative traits

| Phenotypic class | Lower limit^1^ | Upper limit |
| --- | --- | --- |
| 1 | $min$ | $\leq min+{range}/6$ |
| 2 | $min+{range}/6$ | $\leq min+{2x range}/6$ |
| 3 | $min+{2x range}/6$ | $\leq min+{3x range}/6$ |
| 4 | $min+{3x range}/6$ | $\leq min+{4x range}/6$ |
| 5 | $min+{4x range}/6$ | $\leq min+{5x range}/6$ |
| 6 | $min+{5x range}/6$ | $\leq max$ |

^1^min and max: minimum and maximum of the trait in the complete collection, respectively; range: range of the trait in the complete collection
