# Supplementary material for: Development of cassava core collections based on morphological and agronomic traits and SNPS markers
Source: Front Plant Sci. 2023 Sep 6;14:1250205. doi: 10.3389/fpls.2023.1250205 (PMC10511765; doi:10.3389/fpls.2023.1250205)
Supplement: Supplementary file 1 [file DataSheet_1.zip › Table 5 (12).DOCX]

**Supplement**

**Table S5.** The Kappa index calculated to assess the agreement among different methodologies employed for forming core collections, as well as the consolidated collection developed by selecting accessions that were chosen by at least two of the approaches.

| Kappa | GenAN | GenEN | PhenAN | PhenEN | GPmAN | GPmEN | CCons |
| --- | --- | --- | --- | --- | --- | --- | --- |
| GenAN | 1 | -0.07 | 0.02 | 0.05 | 0.11 | 0.01 | 0.19 |
| GenEN | -0.07 | 1 | -0.04 | 0.04 | -0.05 | 0.09 | 0.08 |
| PhenAN | 0.02 | -0.04 | 1 | -0.08 | 0.19 | -0.09 | 0.10 |
| PhenEN | 0.05 | 0.04 | -0.08 | 1 | -0.08 | 0.48 | 0.44 |
| GPmAN | 0.11 | -0.05 | 0.19 | -0.08 | 1 | -0.09 | 0.20 |
| GPmEN | 0.01 | 0.09 | -0.09 | 0.48 | -0.09 | 1 | 0.43 |
| CCons | 0.19 | 0.08 | 0.1 | 0.44 | 0.20 | 0.43 | 1 |

GenAN and GenEN - core collection formed by genotypic data and optimization strategy average accession-to-nearest-entry (AN) and average entry-to-nearest-entry (EN), respectively; PhenAN and PhenEN - Core collection formed by phenotypic data and optimization strategy AN and EN, respectively; GPmAN and GPmEN - Collection formed by morpho-agronomic data + SNPs and optimization strategy AN and EN, respectively; CCons - consolidated collection that includes accessions selected by at least two of the previous approaches.
