# Supplementary material for: Development of cassava core collections based on morphological and agronomic traits and SNPS markers
Source: Front Plant Sci. 2023 Sep 6;14:1250205. doi: 10.3389/fpls.2023.1250205 (PMC10511765; doi:10.3389/fpls.2023.1250205)
Supplement: Supplementary file 1 [file DataSheet_1.zip › Table 6 (6).DOCX]

**Supplement**

**Table S6.** Mean and variance for 15 quantitative traits evaluated in the entire cassava collection and different core collections formed based on phenotypic data (Phen), genotypes (Gen) and pooled phenotypic and genotypic data (GPm) in 1,486 cassava accessions

| **Trait** | **Complete** | | **CCons** | | **GenAN** | | **GenEN** | | **GPmAN** | | **GPmEN** | | **PhenAN** | | **PhenEN** | |
| --- | --- | --- | --- | --- | --- | --- | --- | --- | --- | --- | --- | --- | --- | --- | --- | --- |
|  | Mean | Var | Mean | Var | Mean | Var | Mean | Var | Mean | Var | Mean | Var | Mean | Var | Mean | Var |
| Length of leaf lobe | 14.75 | 08.46 | 14.86 | 9.61 | 14.89 | 10.18 | 14.69 | 07.75 | 14.77 | 07.22 | 15.03 | 11.85 | 14.30 | 05.77 | 14.95 | 11.93 |
| Width of leaf lobe | 03.99 | 01.07 | 4.00 | 1.27 | 04.07 | 01.14 | 03.77 | 01.16 | 03.96 | 00.93 | 04.01 | 01.56 | 04.02 | 00.76 | 03.92 | 01.31 |
| Length and width ratio leaf lobes | 03.92 | 01.59 | 4.01 | 2.40 | 03.88 | 01.65 | 04.22 | 02.42 | 03.94 | 01.35 | 04.09 | 02.73 | 03.70 | 00.88 | 04.11 | 02.29 |
| Petiole length | 21.19 | 26.16 | 21.31 | 32.20 | 21.34 | 34.19 | 20.28 | 21.88 | 21.34 | 20.56 | 21.40 | 40.06 | 20.90 | 20.47 | 21.17 | 40.48 |
| Thickness of the root cortex | 02.39 | 01.04 | 2.36 | 0.41 | 02.33 | 00.41 | 02.38 | 00.52 | 02.45 | 00.36 | 02.47 | 02.91 | 02.37 | 00.35 | 02.46 | 04.21 |
| Root length | 26.17 | 30.17 | 25.92 | 38.31 | 26.21 | 35.64 | 25.60 | 30.10 | 25.81 | 21.29 | 26.06 | 42.85 | 26.24 | 17.39 | 25.96 | 42.51 |
| Root diameter | 58.96 | 79.31 | 59.57 | 103.95 | 59.33 | 72.60 | 58.35 | 61.18 | 59.82 | 70.80 | 58.69 | 125.90 | 59.02 | 66.55 | 59.30 | 135.0 |
| Cyanide content | 06.27 | 02.53 | 6.49 | 2.40 | 06.38 | 02.50 | 06.66 | 02.17 | 06.55 | 02.53 | 06.44 | 02.36 | 06.20 | 02.46 | 06.33 | 02.48 |
| Number of roots per plant | 04.01 | 00.38 | 3.99 | 0.39 | 04.06 | 00.42 | 04.05 | 00.43 | 03.99 | 00.33 | 03.99 | 00.46 | 04.00 | 00.31 | 04.01 | 00.51 |
| Dry root yield | 05.62 | 01.03 | 5.54 | 1.04 | 05.68 | 00.99 | 05.48 | 00.90 | 05.65 | 00.91 | 05.53 | 01.32 | 05.68 | 00.88 | 05.51 | 01.28 |
| Dry matter content | 34.89 | 02.87 | 34.55 | 3.01 | 34.95 | 02.49 | 34.76 | 02.69 | 35.10 | 02.49 | 34.42 | 03.72 | 35.03 | 02.08 | 34.33 | 03.58 |
| Fresh root yield | 17.42 | 24.70 | 17.31 | 27.81 | 17.86 | 24.07 | 16.83 | 21.34 | 17.72 | 21.72 | 17.14 | 32.01 | 17.60 | 20.50 | 17.24 | 33.45 |
| Shoot yield | 19.67 | 23.27 | 18.96 | 22.35 | 19.68 | 22.66 | 19.26 | 24.33 | 19.60 | 21.45 | 19.17 | 27.22 | 19.22 | 18.80 | 18.67 | 28.04 |
| Hasvest index | 45.28 | 45.95 | 45.82 | 56.41 | 45.54 | 49.48 | 44.76 | 46.58 | 45.76 | 45.09 | 45.34 | 67.00 | 46.31 | 31.43 | 46.21 | 66.19 |
| Plant height | 01.92 | 00.04 | 1.91 | 0.05 | 01.94 | 00.05 | 01.91 | 00.04 | 01.93 | 00.05 | 01.89 | 00.06 | 01.92 | 00.04 | 01.91 | 00.05 |

Complete collection of cassava germplasm (Complete), GenAN and GenEN - core collection formed by genotypic data and optimization strategy average accession-to-nearest-entry (AN) and average entry-to-nearest-entry (EN), respectively; PhenAN and PhenEN - Core collection formed by phenotypic data and optimization strategy AN and EN, respectively; GPmAN and GPmEN - Collection formed by morpho-agronomic data + SNPs and optimization strategy AN and EN, respectively; CCons - consolidated collection that includes accessions selected by at least two of the previous approaches.
