# Supplementary figures and images for: Development of cassava core collections based on morphological and agronomic traits and SNPS markers
Source: Front Plant Sci. 2023 Sep 6;14:1250205. doi: 10.3389/fpls.2023.1250205 (PMC10511765; doi:10.3389/fpls.2023.1250205)

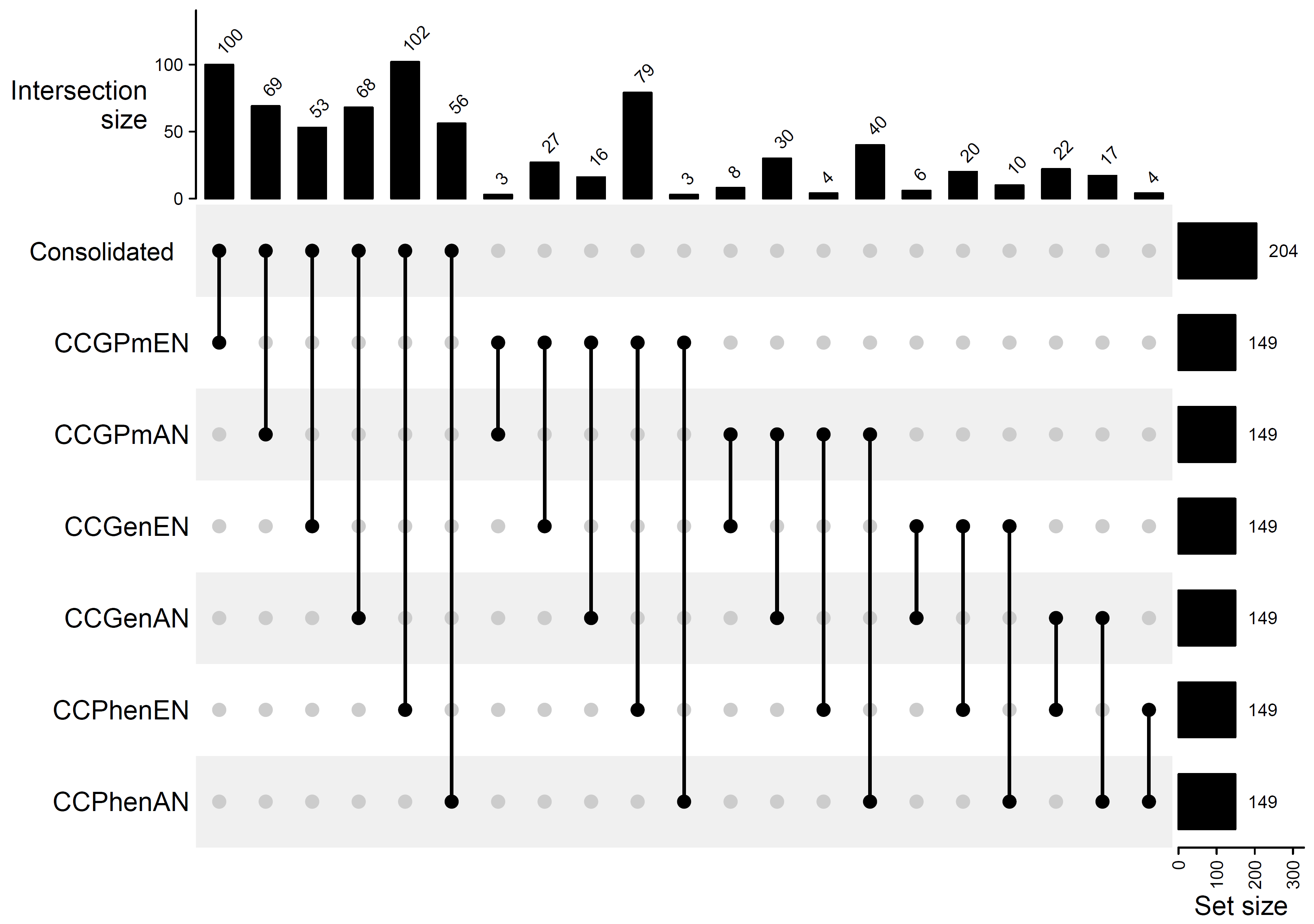

Supplement: Supplementary file 1 [file DataSheet_1.zip › Image 1 (38).TIF]
